# Clinical and Diagnostic Significance of Lactate Dehydrogenase and Its Isoenzymes in Animals

**DOI:** 10.1155/2020/5346483

**Published:** 2020-06-15

**Authors:** Robert Klein, Oskar Nagy, Csilla Tóthová, Frederika Chovanová

**Affiliations:** Clinic of Ruminants, University of Veterinary Medicine and Pharmacy in Košice, Komenského 73, 041 81 Košice, Slovakia

## Abstract

Lactate dehydrogenase (LDH) is widely distributed enzyme in cells of various living systems where it is involved in carbohydrate metabolism catalyzing interconversion of lactate and pyruvate with NAD^+^/NADH coenzyme system. Cells of tissues are direct source of lactate dehydrogenase isoenzymes that are naturally distributed in blood plasma/serum of animals and humans producing characteristic profile. This profile depends on intracellular isoenzyme concentration in all tissues that contribute to the common pool of lactate dehydrogenases in plasma/serum as a consequence of natural cell degradation. LDH is widely distributed in the body, high activities are found in the heart, liver, skeletal muscle, kidney, and erytrocytes, whereas lesser amounts are found in the lung, smooth muscle, and brain. Because of its widespread activities in numerous body tissues, LDH is elevated in a variety of disorders. There are many conditions that contribute to increased activity of LDH. An elevated total LDH value is a rather nonspecific finding. Therefore, LDH assays assume a more clinical significance when separated into isoenzyme fractions. The activity of LDH and its serum and tissue patterns and composition show great variations between the species. These differences do not allow using catalytic activities of LDH isoenzymes from one species to another. Instead, the pattern of serum LDH isoenzymes should be interpreted in respect to its species origin that is important in particular in veterinary medicine. Determination of total LDH activity and its isoenzyme pattern in serum of mammals had become one of the biochemical indicators in the assessment of organ disorders. When the content of cells is released from tissue to plasma, as on cell injury, the LDH isoenzyme pattern of the serum changes in favour of the profile of the affected organ (tissue) that can be used in the diagnostic practice.

## 1. Introduction

The use of biomarkers in medicine lies in their ability to detect disease and support diagnostic and therapeutic decisions. They have also a potential value as an important prognostic tool. Clinically useful biomarkers can supplement the clinical diagnosis and help monitoring of the disease, evaluation of treatments, and predicting prognosis and health outcome. Changes in plasma or serum enzymes and isoenzymes are useful indicators of tissue damage in many diseases. Enzyme increases are usually related to their leakage from damaged cells. In humans, the activity and release of a large body of inflammatory mediators and markers of cell damage such as lactate dehydrogenase (LDH, EC 1.1.1.27) have been evaluated as prognostic and monitoring tools of the disease development, activity, and progression [1]. Cytoplasmatic cellular enzymes, like lactate dehydrogenase in the extracellular space, despite no further metabolic function in this space, are still of benefit because they serve as indicators suggestive of disturbances of the cellular integrity induced by pathological condition. Since LDH is an enzyme present in essentially all major organ systems, serum LDH activity is abnormal in a large number of disorders. Many literature sources and reports indicate that the activity of LDH and its isoenzyme patterns show a great variation between animal species and tissue distribution as well [2–7]. Therefore, it is necessary to understand the composition and distribution of tissue isoenzymes of each animal. The extracellular appearance of LDH is used to detect cell damage or cell death [8, 9]. Increased levels are found in cardiac, hepatic, skeletal muscle and renal diseases, as well as in several haematological and neoplastic disorders. The highest levels of total LDH are seen in pernicious anemia and haemolytic disorders. Liver disorders, such as viral hepatitis and cirrhosis, as well as acute myocardial infarction and pulmonary infarct also show slight elevations of two to three times upper limit of normal. Skeletal muscle disorders and some leukaemias contribute to increased LDH levels. Marked elevations can be observed in most patients with acute lymphoblastic leukemia in particular. Because of the many conditions that contribute to increased activity, an elevated total LDH value is a rather nonspecific finding. LDH assays, therefore, assume more clinical significance if separated into isoenzyme fractions [9].

The fact that the enzyme lactate dehydrogenase is distributed widely in the body required identifying clinical situations in which the determination of lactate dehydrogenase and its isoenzymes in serum are of real value [10]. LDH isoenzyme profiles were the first isoenzyme profiles used in clinical veterinary medicine in an attempt to detect specific organ damage [11]. Even though LDH and its isoenzymes examinations are not used routinely in veterinary laboratory diagnosis, many reports suggested their usefulness in animals. From the clinical perspective, the determination of serum isoenzyme activity is of primary importance, but its determination in biological material from various tissues and organs is also useful [12]. In addition to commonly used blood serum or plasma, LDH activities were also analysed in other body fluids of animals, e.g., synovial fluid [13], cerebrospinal fluid [14], milk [15, 16], and bronchoalveolar lavage fluid [17–19]. There are many reports suggesting their use within experimental investigations, as well as in diagnosis of organ and metabolic diseases, for example, in cattle [3, 20–28].

Examples for disorders and conditions that can cause increased serum activity of LDH are presented in Table 1 [29].

## 2. LDH in Liver and Muscle Diseases

Keller [20] described the behaviour of serum LDH isoenzymes during experimentally induced liver and muscle damage in bovine. Liver damage was induced by administration of CCl_4_ and was followed by considerable increase of LDH_1_, LDH_2_, and LDH_3_ isoenzymes. Total LDH also showed fairly high serum levels, but in view of its normal variation in serum, the changes appeared less significant than that of its isoenzymes. In another experiment, no marked changes were found in total LDH activity during starvation of experimental animals; the only marked change was the rise in LDH_1_ at 7 and 9 days of starvation. After muscle damage produced by making a deep incision into the gluteus muscle among the LDH isoenzymes, the percentage and activity of LDH_5_ showed the most marked increase. The investigation of the effect of local and epidural anesthesia on the activity of serum LDH activity demonstrated greater increase in serum LDH_5_ isoenzyme activity in case of local anesthesia. Thus, local anesthesia appears to cause more damage to muscle tissue than epidural anesthesia. Sanda [30] evaluated the serum enzymes in experimental hepatic lesions and clinical cases with hepatic and muscular lesions in dairy cattle including total LDH activity and its isoenzymes. In calves treated experimentally with carbon tetrachloride with developed hepatic centrilobular congestion, haemorrhage and necrosis the serum LDH activities increased markedly during a 24–49 hour period and the increase reflected the severity of the hepatic lesions. Total serum LDH activity increased with the occurrence of skeletal muscular and hepatic lesions. However, isoenzyme LDH_4_ and LDH_5_ activities were high in the skeletal muscular lesions and low in the hepatic lesions. The author concludes that when the activity of total serum LDH is high, LDH isoenzymes should be assessed. He recommended this enzyme as an aid in differential diagnosis of hepatic and/or skeletal muscular lesions in dairy cattle.

First reports on the possibility of using LDH isoenzymatic separation in disease diagnosis in ruminants appeared in the sixties, when Boyd [31] noted a significant increase in the activity of LDH_5_ in lambs with acute muscular dystrophy. Selected biochemical indicators were investigated by Sobiech and Kuleta [23] in an experiment in lambs with subclinical form of nutritional muscular dystrophy (NMD) to determine their usefulness in early diagnosis in this disease. The authors found higher total LDH activity in experimental group of lambs and, moreover, the greatest variation in the activity was observed with the LDH_5_ fraction as an enzyme specific to the skeletal muscle. The high LDH activity reflects muscle fiber damage and LDH_5_ leakage into the blood. Boyd [31] showed also an increase in LDH_5_ activity when clinical symptoms of the disease were present. There is a lot of information in the literature regarding the usefulness of the determination of LDH activity in the diagnosis of NMD in lambs and goat kids [32, 33]. However, not many investigators support this analysis, because LDH is not specific to the muscular tissue and its total activity increase in muscular dystrophy, liver necrosis, cancer, and various disorders of the digestive system [22, 34] and determination of other enzymes activities should be taken into consideration when diagnosing subclinical states of NMD.

Activities of blood serum LDH were investigated in seven calves during the course of experimental sarcocystosis [35]. Development of muscular cysts was accompanied by degenerative changes and rupture of muscle fibers and sometimes even myositis develops. Sarcocysts can cause also hepatic lesions such as degenerative hepatitis. These processes result in increased catalytic activities of several serum enzymes including LDH and its isoenzymes. Catalytic activity of LDH in calves remained unchanged until the 7th week after infection and was elevated from the 7th week with a maximum in the 9th week after infection. Likewise, the proportion of LDH_1_ isoenzyme showed an increase from the 7th week reaching a maximum in the 9th week. The changes in the LDH isoenzyme spectrum with an increasing liver fraction are indicative of liver damage. The activity of the enzyme correlated with the course of the disease.

Plasma LDH activity is nonspecific for hepatocellular disease in birds. Compared with plasma aspartate transaminase (AST) and alanine transaminase (ALT) activity, plasma LDH activity in birds increases and declines more rapidly after injury to liver or muscle. The short mean elimination half-life of LDH compared to that of creatine kinase (CK) makes it a valuable test in differentiating between muscle and liver disease in pigeon. In most birds, increased plasma LDH activity with normal CK activity is suggestive of hepatocellular disease. The plasma LDH activity is considered to have a wide tissue distribution in reptiles. Therefore increases in the plasma LDH activity may be associated with damage to the liver, skeletal muscle, or cardiac muscle [36].

## 3. LDH and Mastitis

Indirect indicators of mastitis are important for the diagnosis of the disease (mainly subclinical form). Diagnosis of subclinical mastitis is of increasing importance and appropriate detection methods are needed. Subclinical mastitis (SCM) is difficult to detect due to the absence of any visible indications, and it has major cost implications [37]. The usefulness of soluble markers in indirectly differentiating inflamed quarters from healthy quarters was tested in foremilk samples by several authors. They have evaluated milk enzyme activities changes (LDH, ALP, and AST) to diagnose udder infections in dairy cows [15, 16]. According to results presented by Zank and Schlatterer [38], cell-damaging processes during mammary inflammation should best be detected by measuring elevated LDH activity.

The level of total LDH activity in dairy cow milk serum was studied in sets of quarter-udder milks showing different degrees of a positive response to mastitis test by Kováč and Beseda [39]. Increasing positiveness of the response to this test was found to be accompanied by almost proportionate increase of the total LDH activity level in milk serum. The bacteriological finding was in this study not correlated with increasing positiveness of the response to California mastitis test. Bogin and Ziv [40] have already suggested that LDH in milk is sensitive indicator of epithelial cell damage and subsequently proposed that LDH originated mainly from the damaged udder epithelial cells and from the elevated numbers of leukocytes. Thus this parameter might be suitable for use in the early diagnosis of SCM in cows. Bogin et al. [15] determined the pattern of distribution of LDH isoenzymes in normal and inflamed bovine udder tissues, in normal and mastitic milk leukocytes and serum. LDH_1_ was the most common isoenzyme found in all the types of tissues examined; the inflamed tissues and leukocytes from mastitic milk showed a higher proportion of LDH_4_ and LDH_5_. It seems that the origin of the elevated LDH in mastitic milk is the leukocytes and the parenchyma cells of the udder. Hiss et al. [41] described the quantification of LDH in milk samples from healthy and subclinically diseased udder quarters. Authors found positive correlation between somatic cell count (SCC) and LDH activity in milk and this is useful parameter for the diagnosis of subclinical mastitis. Healthy quarters could be differentiated from quarters suffering from subclinical mastitis. However, udder quarters with latent infections without elevated SCC could not be distinguished from the healthy quarters using LDH activity. Yang et al. [42] evaluated the changes occurring in milk malondialdehyde level and some enzymatic activities as a result of subclinical mastitis in dairy cows. The mean level of LDH activities was significantly higher in SCM milk than in normal milk. They stated that the measurement of this parameter in milk appears to be a suitable diagnostic method for identifying SCM in dairy cows. Chagunda et al. [43] studied the systematic factors affecting the activities of LDH and N-acetyl-beta-D-glucosaminidase (NAGase) and somatic cell count (SCC), the association between the activities of LDH and NAGase and SCC with respect to udder health status, and the ability of LDH and NAGase to classify cows into udder health categories for early detection of mastitis. All the three parameters increased due to clinical mastitis. LDH activity had higher sensitivity than NAGase activity and sensitivities for LDH activity were more robust to changes in the threshold value than those for NAGase activity. In an experiment conducted by Vyavahare et al. [44], the changes in the activity of LDH in the milk-whey and blood plasma associated with udder health status of cows were investigated. The LDH activity in milk-whey differed significantly among different udder health statuses, but it did not differ significantly in blood plasma. They concluded that the alterations in LDH activity in milk-whey are thus useful to assess the udder health status of the cows. Mohammadian [45] assessed the relationship between LDH activity and SCM occurring naturally on dairy herds. LDH was analysed in blood serum and milk. According to the results, the mean activity of LDH was higher in milk from udders affected with SCM than in milk from healthy udders. There were no significant differences in blood serum LDH of healthy and SCM cows. The higher level of LDH in mastitic milk than blood serum shows that blood serum was not the sole source of this enzyme in mastitic milk and it was probably also liberated from disintegrated leukocytes and the parenchymal cells of the udder. The increment in LDH in milk of udders shows the presence of tissue damage provoked by SCM. The origin of LDH in mastitic milk is attributed to leukocytes and also epithelial cells from the udder [15, 46].

The relationship between somatic cell counts and LDH activity in milk was examined by Nizamlioglu and Erganis [47] to find out the suitability of these variables for early detection of subclinical mastitis in Merino ewes. A significant positive correlation was found between LDH activity and SCC in ewes' milk. Activity of LDH was significantly higher in milk from inflamed udders than in normal milk. LDH activity in milk samples appeared to be a sensitive and specific indicator of subclinical mastitis. Blood constituents were measured in the blood serum from healthy and mastitic cows by Atroshi et al. [48]. The LDH activity was higher in mastitic than in healthy cows. The increase in LDH activity in mastitic animals may reflect the increased enzyme activity associated with inflammatory changes or trauma to the udder tissue. An increase in LDH concentration may reflect both selective release due to the inflammatory stimulus and nonspecific tissue cells or leukocyte death [49]. Furthermore, it has been reported that the increase in LDH activity during experimental mastitis in goats reflected the degree of inflammation [50].

Several studies and literature data showed that the mean LDH activities in the milk from udders with SCM were significantly higher than in that from normal udders and activities were significantly higher with higher California mastitis test scores. Since the blood-milk barriers are damaged with infection (inflammation), it is also possible that LDH was transferred from blood to the milk. However, Batavani et al. [51] showed that blood serum was not the sole source of this enzyme in ovine mastitic milk and that it was probably liberated from udder parenchymal cells and from disintegrated leukocytes [46, 52].

## 4. LDH in Respiratory Diseases

Respiratory diseases are common and serious health problem in animals that often affect the upper and lower airways. However, in animals there are limited blood biochemistry variables useful for the laboratory analysis of the degree and extent of the lung damage. According to the present knowledge, mainly from human medicine, LDH is one of these potential parameters presented in the literature as a possible indicator of the lung damage [53]. These authors reviewed the usefulness of monitoring the activity of LDH and its isoenzyme pattern as possible indicators of pathological conditions in the lungs, such as cell damage or inflammation and several studies have shown the diagnostic use of the measurement of LDH activity in pulmonary diseases. Lung-related disorders include obstructive, microbial, interstitial, and other pulmonary diseases. It has been suggested that increased plasma LDH_3_ isoenzyme activity reflects acute lung injury causing cell damage and cell death, as LDH_3_ was to be found elevated in the plasma when pulmonary embolism occurred. This isoenzyme may be useful biochemical index of acute immunological antibody mediated lung injury, with potential diagnostic and prognostic value in pulmonary disease [54]. Although the increase in total serum LDH activity is rather nonspecific, it is proposed that measurement of LDH activity levels and its isoenzyme pattern in pleural effusion and, more recently, in bronchoalveolar lavage fluid may provide additional information about lung and pulmonary endothelial cell injury [53].

However, there are scarce sources in the literature regarding the activity of LDH and its isoenzymes in animals suffering from respiratory diseases. In ovine inoculated with parainfluenza virus type 3 and *P. haemolytica*, Davies et al. [17] found in bronchoalveolar lavage (BAL) fluid increased levels of LDH reflecting probably increased cell damage. Weiss et al. [18] in calves with experimentally induced pneumonic pasteurellosis detected in lavage fluid increases of LDH activities. Increased total activity of LDH in lavage fluid in calves obtained from pneumonic lesions was recorded also by Reinhold et al. [19] and the levels of LDH activity positively correlated with the severity of the lung damage. Bhat et al. [55] studied alterations in the serum LDH activity of *Dictyocaulus filaria* infected lambs and found significant increase in its activity during patency. These changes correlated well with the progress of the disease and lung damage caused by the parasite. They suggested that the elevated LDH activity is the direct result of lung damage caused by the parasite. Thus monitoring LDH levels being a nonspecific enzyme may be of some use assessing lung damage and in conjunction with clinicoparasitological and pathological observations may be of significant help in determining the therapeutic value of an anthelmintic.

Lung tissue, like other organ tissues, is rich in LDH and the tissue levels are about 500-fold higher than those normally found in serum [53, 56]. This should be the assumption for its release from the damaged cells and elevation of blood activity of LDH and changes in proportion of isoenzyme patterns. Milne and Doxey [57] measured the total LDH activity and the percentage levels of its isoenzymes in lung lesion from lambs with acute and chronic pneumonia and found higher total enzyme activity mainly with increases of activity of the LDH_4_ and LDH_5_, particularly in chronic pneumonia. They consider lung lesion as a potential for altering the serum isoenzyme distribution. In human medicine, elevations of serum LDH_3_ occur most frequently with pulmonary involvement and have been observed in patients with various carcinomas as well [9]. Elevation of LDH_3_ isoenzyme was found by Hagadorn et al. [54] in rat after experimentally induced immunologic lung injury. These authors suggest that LDH_3_ isoenzymes are released into the circulation from the cells in the damaged lung. In horses suffering from respiratory diseases, Sommer et al. [58] investigated the serum activity of enzymes and found increased activity of LDH. Georgiev and Monov [59] reported an increase in total serum LDH and a considerable rise mainly in the activity of the isoenzymes LDH_1_ and LDH_2_, which they considered to be characteristic for pigs affected with bronchopneumonia. As blood samples more commonly are used in animals for laboratory analyses, Nagy et al. [60] investigated the possible usefulness of this enzyme in blood serum as an indicator of lung damage. In calves with clinical signs of lower respiratory tract diseases, they found significantly higher total activity of LDH than in clinically healthy animals. In contrast to the results presented in humans or other species of animals, the changes in isoenzyme pattern are characterized by remarkable increase of the LDH_1_ fraction and significant decrease in the proportion of the other four LDH isoenzymes. They found a noticeable shift of the isoenzymatic activity towards the LDH_1_ fraction. Increase in LDH activities indicated possible injury to the respiratory tract epithelium and release of LDH from epithelial cells lining the airways, which should be an indicator of damage to those cells. Inflammation of the lung is followed by influx of polymorphonuclear cells and activation of alveolar macrophages as well as with the changes of the alveolar-capillary barrier permeability [53]. The specificity of LDH isoenzyme patterns of these infiltrating cells could also be responsible for the species differences in the changes of LDH isoenzymes distribution. This assumption could be indicated by the results of LDH isoenzyme patterns in cow peripheral leukocytes [26, 61].

## 5. LDH in Relation to Metabolic Diseases, Feeding System, and Milk Production

Animal species differ in their nutritional metabolism. This is reflected in the differences in their plasma metabolite concentrations [62]. Activities of enzymes related to energy metabolism and concentrations of metabolites as substrates or end-products of the energy metabolism may become important parameters for evaluating the metabolic states of animals [63]. One of the indices determined in the diagnostics of metabolic diseases is the total activity of lactate dehydrogenase and its isoenzymes.

Biochemical alterations in serum and cerebrospinal fluid in experimental acidosis in goats were studied by Lal et al. [64]. They observed significantly higher activity of LDH in serum samples of acidotic animals. Asefa Asmare et al. [65] analysed blood serum to investigate the total activity of LDH and LDH isoenzymes in dairy cows at different stages of milk production. They found that the total activity of LDH was higher in late lactating cows followed by early lactating and late pregnant cows. In late pregnant cows, the serum activity of LDH_1_ isoenzyme was found to be significantly higher than in early lactating cows. In all groups of animals, no statistically important variation in the activities of LDH_2_ and LDH_3_ isoenzymes was observed. The significantly elevated serum activity of LDH_4_ isoenzyme was recorded in early lactating cows. Percentual serum distribution of LDH_5_ isoenzyme was found to be significantly increased in early lactating cows compared to late lactating cows and cows in late pregnancy. These findings implied that, in sera from early lactating cows, the activities of liver isoenzymes LDH_4_ and LDH_5_ were much more prominent than in sera from late lactating and late pregnant cows and that the serum activities of LDH isoenzymes varied according to the stages of milk production. According to Bauman and Bruce Currie [66] during the early lactation period, high-yielding cows can be in negative energy balance and metabolic diseases with impact on the liver function most commonly occur during this period with changes of the activity of LDH and LDH isoenzymes.

Investigations of the influence of increased intake of fats in early lactation period on the development of liver steatosis in cows showed that, besides other blood biochemistry indices two weeks postpartum, the experimental group of cows had a significantly higher blood serum activity of LDH as a result and confirmation of the development of liver steatosis [25]. The increase of LDH activity was related to an increase in the degree of steatosis [67]. On the other hand, Asefa Asmare et al. [68] found no significant changes in serum LDH activity linked to liver damage, although they were able to demonstrate a degree of influence in a study of LDH isoenzymes. Activities of enzymes related to energy metabolism were measured in plasma of Korean and Japanese beef cattle raised by different feeding systems by Mori et al. [69]. LDH activities in the plasma of Korean beef cattle were significantly higher than those of Japanese beef cattle. New Zealand beef cattle fed on pasture which they harvest by grazing showed significantly lower LDH activity than in Korean and Japanese beef cattle fed on larger amount of concentrate diets. The higher activity of plasma LDH may indicate slight liver damage by slightly acidotic conditions in these animals and accelerated function of liver for more nutrients' metabolism. Mori et al. [27] measured several blood parameters of pregnant Angus heifers with differing liveweight change profiles (gaining or losing) to investigate their meanings in the restricted feeding beef heifers. There were no significant differences found between the groups of animals in plasma LDH activities. The composition ratios of LDH_4_ and LDH_5_ in plasma of the liveweight gaining heifers were higher than those of the liveweight losing heifers. Bellová et al. [28] compared the influence of fat supplementation in the form of full-fat soybean seeds and hydrolysed palm oil as energy sources for dairy cows in early lactation during the first 8 lactation weeks and found changes in the LDH activity in blood serum as well as in LDH isoenzymes LDH_1_ and LDH_2_. The results demonstrated a lower extent of liver parenchyma damage in cows with soybean seeds as a source of fat. Hatzipanagiotou et al. [70] examined the effect of the energy level of the feed ration on the LDH isoenzyme in the blood serum of “Thessaloniki” crossbreed type lambs. The effect of the energy level upon the relative distribution of LDH isoenzymes was significant only for the isoenzymes LDH_1_ (higher) and LDH_5_ (lower) in animals with higher energy content in the feed.

Sako et al. [62] measured the LDH activity in dogs, cats, horses, cattle, and sheep to investigate the significance of these parameters in animals with different mechanism of nutrient metabolism and found great differences in plasma LDH activity between species. These differences are considered to be due to differences in the particular characteristics of the species, to use as their main energy source glucose or rumen fermentation products.

## 6. LDH and Intoxications

There is also literature data regarding the effect of some intoxications on the changes of activity of LDH. Boyd [71] induced liver necrosis by carbon tetrachloride given orally in sheep, calves, cows, and rats and compared the activity of some enzymes. LDH produced the highest increase in serum activity in all animals. The increase was greater in those animals, in which hepatic necrosis was most extensive.

Šutiaková et al. [72] investigated the effects of high chlorine levels in drinking water upon the total activity of LDH and its isoenzymes in the blood plasma before and after 30 days' exposure of merino lambs. After a sixfold increase of chlorine levels in the drinking water, significant differences in the total LDH activity, as well as in LDH_2_ (decrease) and LDH_5_ (increase) isoenzyme activity, were observed between the experimental and control groups of lambs. They suggested that LDH activity and its isoenzymes in the blood plasma of animals seem to be suitable biomarkers for ecotoxicological studies. In a similar study, but after short-term administration of chlorine in drinking water, no significant differences were found between the control and experimental group of animals in the total LDH activity and its isoenzymes [73]. In another experiment, Šutiaková et al. [74] studied the activity changes of LDH isoenzymes in the blood plasma of young rams and ewe hoggets in experiments with carbimazole. Out of LDH isoenzymes, only LDH_4_ isoenzyme exhibited significant differences in the blood plasma of ewe hoggets. Haskovič et al. [75] studied the effect of glyphosate on serum enzyme activity in rats. Glyphosate was applied subcutaneously and administrated every 24 hours for a 15-day period. After the administration of glyphosate, the LDH activity significantly rose.

## 7. The Effect of Age, Stress, Exercise, Breed, Sex, and Season on the Activity of LDH

Avallone et al. [76] investigated the variations of LDH activities in young water buffalo calves. Differences in total activities as well as their relative distribution were seen at ages ranging from 1 to 10 weeks. While total LDH activity increased by over 100%, the relative activities of LDH_1_ and LDH_5_ decreased with age. LDH_2_ and LDH_3_ increased and LDH_4_ did not change. Frerking et al. [77] investigated the enzymes in healthy calves and found approximately constant activity of total LDH during the time before colostrum intake till the eighth week of life. In another study in calves, total serum LDH activity was lower at birth than at weaning and yearling and activity of total LDH was similar among 4- to 10-year-old cows [78].

Beatty and Doxey [2] analysed the effect of age, sex, and growth rate on total serum lactate dehydrogenase and its isoenzymes in Suffolk × Half-bred lambs from 1.5 days to 10 weeks old. The total LDH activity was higher at 1.5 days than in older lambs and the percentage of LDH_1_ tended to increase while LDH_4_ and LDH_5_ decreased with age from 2 weeks, indicating that the isoenzyme distribution was changing towards the adult pattern. In female lambs, the percentage of LDH_1_ was higher than in male lambs at 1.5 days, but little importance was attached to this finding since the difference was not significant at 10 weeks. No clear relationships existed between the level of total LDH or its isoenzymes and daily liveweight gain. The age-related changes were considered to be of greater significance than those related to sex and growth rate when interpreting serum LDH levels in lambs. The serum LDH isoenzyme activities are little affected by their sex or growth rate. The age of the lambs is, however, important when the clinical significance of the results is considered. The change in total serum LDH activity and the trend towards the adult serum isoenzyme pattern with increasing age are noteworthy. Looper et al. [79] analysed the relationship of LDH activity with body measurements of beef cows and calves and found that LDH activity seems to be altered in the serum of growing animals, but results from this experiment suggest that LDH activity remains stable in mature cows. According to Koh and Choi [80], lactate dehydrogenase activity may increase in the serum during cell growth and development due to loss of cell membrane integrity. Bide et al. [81] found large seasonal changes in mean plasma LDH in the range cattle but not in confined, constant diet group. The changes corresponded with the appearance of new growth of the range. Changes in the plasma LDH were observed at weaning time in the range cattle but not in the confined group. Sex had no effect upon plasma LDH levels in the juvenile animals of either group. An increase in plasma LDH was observed around the first ovulation in the confined animals and a decrease in plasma LDH was observed during parturition in the confined group.

Sobiech et al. [12] determined and compared serum LDH isoenzymatic profiles in the most common breeds of dairy cattle in Poland and found different total activity of LDH in particular cattle breed. However, they emphasized that the differences noted between the breeds remained within reference values for this species. Serum isoenzymatic separation showed that the activity of LDH_1_ in beef cows was statistically significantly lower than in dairy cows. This could be connected with higher productivity of dairy cows and thus higher liver load, as in ruminants isoenzyme LDH_1_ is present in the cardiac muscle and liver, and changes in its activity reflect the functional condition of these organs. Similarly to the activity of LDH_1_, the activity of LDH_2_ was higher in the serum of dairy cows, whereas the activity of isoenzymes LDH_3_, LDH_4_, and LDH_5_ (especially the last two ones) was considerably higher in beef cows. This is natural, as in ruminants LDH_4_ and LDH_5_ are characteristic of skeletal muscles, and muscle weight is very high in beef cows. This fact should be kept in mind while interpreting LDH separation in cattle in the diagnostics of heart, liver, and skeletal muscle diseases.

Anderson [82] studied the effect of exercise on the serum LDH activity and its isoenzyme pattern and observed after exercise increase of total LDH. Isoenzyme studies on serum before and after exercise demonstrated that most of the increase in serum LDH is attributable to an increase in LDH_4_ and LDH_5_. From tissue LDH patterns, these isoenzymes are most likely to originate from skeletal muscle and/or liver (since skeletal muscle contains little LDH_4_). The overall picture suggests that damaged skeletal muscle is the source of the increase in serum LDH observed after strenuous exercise although the liver may also contribute to the increase in LDH. Goddard et al. [83] described the physiological response to stress in red deer using the assessment of LDH and its isoenzyme LDH_5_, as a marker of muscle damage. After three-hour journey, the study showed increase in LDH activity in plasma due to rise in plasma LDH_5_ activity. They concluded that rise in LDH_5_ isoenzyme activity might be a useful in vivo marker for muscle damage. No changes in total LDH and LDH isoenzymes in muscle and serum were observed in adult Landrace pigs following exercise by Doizé et al. [84].

## 8. LDH in Other Diseases and Conditions

In humans, serum lactate dehydrogenase is used as a prognostic indicator in non-Hodgkin's lymphoma [10]. In veterinary medicine, only a limited amount of information is available regarding the pattern of LDH isoenzymes related to lymphoma. An increase in the value of serum LDH has been reported in dogs affected by leukemia [85] and in cats with large granular lymphoproliferative disorders [86]. Bezzecchi et al. [87] found that an increase in LDH_3_ was possible indicator of latent canine lymphoma. Abate et al. [88] showed that LDH_2_ and LDH_3_ were increased in dogs with lymphoma. Zanatta et al. [89] evaluated the clinical utility of monitoring changes in total and isoenzyme patterns of LDH at diagnosis of canine lymphoma and during the course of therapy. In all disease groups of animals, they found nonstatistically significant increase in total serum LDH values. They suggested that the increase of LDH_2_ and LDH_3_ in dogs with enlarged lymph nodes can be considered to be diagnostic indicator of lymphoma. Moreover, determination of the isoenzyme pattern also provides information about the therapeutic response. For dogs in remission, the isoenzymatic patterns were comparable to that found in healthy animals. At diagnosis, the survival time was significantly higher for dogs with lower levels of serum LDH.

Dawra et al. [22] studied the enzymatic profile of urine and plasma in field cases of bovine bladder cancer. Urinary LDH activity was significantly higher and the isoenzyme pattern revealed elevated LDH_2_ and LDH_4_ in the urine from affected animals. In plasma, LDH activity was significantly elevated without any change in the isoenzyme pattern.

The objective of the study presented by Hoogmoed et al. [90] was to determine whether selected parameter analysed in peritoneal fluid including LDH activity can be used to differentiate horses with septic peritonitis from horses with nonseptic peritonitis. They found that the mean serum LDH activity in horses with nonseptic peritonitis was significantly higher than in horses with septic peritonitis and in healthy horses. Horses with septic peritonitis had higher LDH activities in peritoneal fluid than in serum. Horses with nonseptic peritonitis did not have significant differences in LDH activities between peritoneal fluid and serum. The high mean serum LDH activity in horses with nonseptic and septic peritonitis may reflect absorption from the peritoneal fluid [91]. Peritoneal fluid LDH activity may be useful in the diagnosis of sepsis in the nonsurgical horse, but it does not appear to be a valuable diagnostic aid of the postsurgical horse because significant differences were not found in the peritoneal fluid LDH activities between the horses with septic and nonseptic peritonitis. LDH activity in equine pleural and synovial fluid has been also used to detect sepsis with the potential advantages of speed, ease of measurement, and lower cost relative to bacterial cultures [92, 93].

Allwin et al. [94] analysed haematobiochemical parameters as prognostic indicators in elephant colic. Among the several parameters, LDH was found to be significantly elevated in elephants with moderate and severe colic. This was in accordance with the report of Sabev and Kanakov [95] who reported extremely elevated LDH activity in elephants with caecal impaction. Sobiech and Kuleta [96] evaluated selected blood biochemistry parameters in calves during the course of diarrhoea. In sick calves, they observed insignificant, slightly higher total LDH activity and a significant increase in LDH_1_ activity together with a decrease in LDH_5_ isoenzyme activity. Similar results were obtained while investigating changes in the isoenzyme profile of LDH in the serum of diarrhoea-suffering goat kids [97].

In Thoroughbred horses with histories of mild locomotory disturbances and/or poor performance, increased skeletal muscle membrane leakage was signalized by elevated activity of LDH and changes in isoenzyme distribution. LDH_4_ and LDH_5_ were significantly increased whilst LDH_1_ and LDH_2_ decreased [98].

Biochemical studies have shown that LDH isoenzymes are present in uterine secretions and that LDH activities vary with the stage of the estrous cycle [99]. Wright and Grammer [100] evaluated serum LDH isoenzyme patterns in open and pregnant Holstein and Hereford cows as a method of detecting pregnancy. LDH_4_ and LDH_5_ were found in higher concentration in pregnant versus open Holstein cows and no differences were found in serum LDH isoenzyme patterns between pregnant and open Hereford cows. However, differences in concentration of all five LDH isoenzymes were observed between Holstein and Hereford cows. The results suggest that elevation of serum LDH_4_ and LDH_5_ may be used as a means of pregnancy detection in the Holstein but not the Hereford cow.

Peripartal serum LDH activity was measured in dairy cows in order to examine the association between retained fetal membranes (RFM) and enzyme activity [101]. LDH activities in nonretained and retained placenta cows were similar. LDH data showed a moderate increase in activities starting from day 3 before parturition until day 3 postpartum in both groups of cows, but the difference was not significant. However, the differences in LDH activities on different days within nonretained and retained placenta cows were significant. Results indicate that prepartal changes in LDH activities are not predictive of placental retention postpartum. These increased serum LDH activities during the peripartum period in both groups are in contrast to a study presented by Dutta and Dugwekar [102]. In their study, higher prepartum serum activities of LDH were demonstrated in cows which subsequently retained fetal membranes after parturition. The results revealed that the levels of LDH were significantly higher in cows with RFM during late gestation and continued to be higher until 5th day postpartum. Thus, it seems probable that prepartum increases in LDH activities could be useful indicator of the presence of uterine and placental pathology. The reason for the rise in peripartum LDH activities is open to speculation. It is noteworthy that LDH activity increased dramatically in the amniotic fluid just before bacterial induced fetal death and abortion, without comparable changes in LDH activity in the maternal plasma [103]. This observation suggests that the fetoplacental tissues are a source of LDH.

## 9. Conclusions

Analysis of total LDH activity and predominantly its isoenzyme pattern can significantly contribute to the diagnosis of diseases, which are linked to tissue damage. Identification of isoenzymes that are causing the increase in total LDH activity could increase the diagnostic value of LDH activity. However, isoenzyme analysis requires special assays that are not routinely and widely available in each laboratory, and species variations in tissue distributions make interpretations difficult. By itself, serum LDH activity is a screening test for some tissue damage and, in a group of tests, LDH activity provides additional information that may help explain activities of more tissue-specific enzymes. Presented data points out that analysis of activity of LDH and its isoenzymes has clinical and diagnostic importance in laboratory diagnosis in animals. Knowing the physiological differences and specifics of each animal species forms the basis for their implementation and use in the veterinary clinical pathology. Even though LDH and its isoenzymes examinations are not routinely used in veterinary laboratory diagnosis, many reports suggested their usefulness in the diagnostics of animal diseases.

## Figures and Tables

**Table 1:** Disorders or conditions that can cause increased serum LDH activity (adapted from Stockham and Scott).

*Hepatocyte damage*
(i) Degenerative—hypoxia due to anemia or congestion and cholelithiasis
(ii) Metabolic—lipidosis or fat cow syndrome, diabetes mellitus, and equine hyperlipidemia
(iii) Neoplastic—lymphoma and metastatic neoplasia and hepatocellular carcinoma
(iv) Inflammatory
(a) infectious—bacterial and necrotic hepatitis, cholangiohepatitis, and hepatic abscess
(b) noninfectious—Theiler's disease, chronic hepatitis, and cirrhosis
(v) Toxic—iron toxicity, alkaloid-containing plants, and aflatoxins
*Muscle damage*: skeletal or cardiac muscle (mostly skeletal)
(vi) Degenerative—hypoxia due to exertion or seizures, exertional rhabdomyolysis, and saddle thrombus
(vii) Metabolic and nutritional—feline hyperthyroidism and vitamin E or selenium deficiency
(viii) Neoplastic—metastatic neoplasia
(ix) Inherited—musculodystrophy and hyperkalemic myopathy
(x) Inflammatory—myositis (*Neospora and Toxoplasma*), bacteria, or other agents
(xi) Toxic—monensin, castor bean, and gossypol
(xii) Traumatic—intramuscular injection, hit-by-car, recumbency, seizures, and exertion
*Haemolysis* (or prolonged serum contact with erythrocytes)
